# Stakeholder Perspectives of the Inflation Reduction Act’s (2022) Impact on Prescription Drugs: A Narrative Review

**DOI:** 10.3390/pharmacy11060187

**Published:** 2023-12-17

**Authors:** Cristian Lieneck, Matthew McLauchlan, Valerie Adachi, Roger Billings

**Affiliations:** 1 School of Health Administration, Texas State University, San Marcos, TX 78666, USA; 2 OttoBock HealthCare, Austin, TX 78758, USA; jmatthew.mclauchlan@ctx.edu; 3 Cambridge College Global, Boston, MA 02109, USA; valerie.adachi02@go.cambridgecollege.edu; 4 Salmon P. Chase College of Law, Northern Kentucky University, Highland Heights, KY 41099, USA; billingsr@nku.edu

**Keywords:** Inflation Reduction Act, prescription drugs, pharmaceuticals, drug prices, inflation

## Abstract

In this review, we examine the impact of the Inflation Reduction Act (IRA) of 2022 on pharmaceutical drugs in the United States, drawing on a diverse range of sources to understand the perceptions of multiple stakeholders and professionals. Findings suggest that the Act, while aiming to control price inflation, has had a multifaceted impact on the pharmaceutical sector. Stakeholders, including pharmaceutical companies, healthcare providers, patient advocacy groups, and policymakers, offered varied perspectives: while some laud the Act for its potential in controlling runaway drug prices and making healthcare more accessible, others raise concerns about possible reductions in drug innovation, disruptions to supply chains, and the sustainability of smaller pharmaceutical companies. The review identified four underlying constructs (themes) in the literature surrounding healthcare stakeholders’ perceptions of the IRA’s impact upon prescription drugs: pricing and/or dictation pricing issues, topics related to patent law and pharmaceuticals, processes surrounding the IRA’s (2022) rules and regulations, and potential threats to the pharmaceutical industry concerning the research and development of future medications. The complex interplay of the Act’s implications underscores the importance of ongoing assessment and potential iterative policy refinements as implementation endures.

## 1. Introduction

The rising cost of prescription drugs has become a significant concern within the healthcare sector, impacting patients, healthcare providers, insurers, and the United States government. In response to this issue, policy measures aimed at curbing inflation and reducing healthcare expenditures continue. One such policy initiative is the Inflation Reduction Act (IRA) of 2022, a legislative intervention designed to address escalating costs within the pharmaceutical industry, patient affordability of prescription drugs, and reduce Medicare drug expenditures [1]. An important goal of the Inflation Reduction Act of 2022 is to reduce prices for brand-named drugs in the United States. At first glance, the IRA seems to cut the prices of only a few drugs. Ten will be selected for 2026, fifteen more for 2027 and 2028, twenty for 2029, and twenty each year thereafter. Any of those will have been on the market for at least nine years (thirteen for biologicals) and priced during that time at the manufacturers’ discretion. Even after seven years, manufacturers are supposed to have input on price control decisions. Finally, only the drugs used in Medicare are subject to price controls.

This narrative literature review seeks to comprehensively analyze and synthesize the existing body of research and publications regarding the impact of the IRA on prescription drugs in the United States, as perceived by various healthcare industry stakeholders in the recently published literature. Understanding the potential impact and outcomes of the Inflation Reduction Act is essential for shaping future healthcare policies aimed at ensuring affordable and accessible prescription drugs for all. This review aimed to provide a comprehensive understanding of the effectiveness and implications of the IRA in addressing the economic and healthcare challenges associated with escalating prescription drug prices. Due to the recentness of the IRA and its application, limited research has been completed to date.

### 1.1. Rationale

A burst of articles has already appeared around the effects of the IRA on Medicare costs, on the one hand, and manufacturer profits, on the other. Politicians have long criticized what they perceive as the overpricing of patented drugs, but have always been blocked to affect pricing by the manufacturers—until now. Although the IRA application has recently begun, its impact has been considered a potential paradigm shift by multiple stakeholders with opposing expected outcomes. However, all expect it to open the door for the broader control of drug pricing and industry change. Fearing this will be the case, major drug manufacturers have filed lawsuits to block its implementation [1]. They argue that it violates their constitutional and statutory rights, and they challenge the penalties of more than USD 500 million that can be imposed on a manufacturer for refusing to negotiate price reduction.

### 1.2. Purpose

Hospitals, providers, and Medicare beneficiaries are waiting to see how the IRA will affect health care. The narrative review was conducted to query current literature focused on the Act’s influence on pharmaceuticals in the U.S., as projected by multiple healthcare industry stakeholder types. Findings will continue to support the understanding and development of ongoing/future U.S. policy and procedures surrounding prescription drugs for the U.S. healthcare system.

## 2. Methods

### 2.1. Overview

The research team’s initiative was to specifically identify various stakeholder perspectives surrounding the 2022 Inflation Reduction Act policy regarding prescription drugs in the United States as identified in the literature review process. The PRISMA (preferred reporting items for systematic reviews and meta-analyses) review standard guided the research team’s review process.

Two separate university library EBSCOhost research platforms were used in the initial review process to identify as many potential articles as possible for the review: Texas State University and Concordia University–Texas. This unique approach was decided on by the research team after identifying the limited information published to date in peer-reviewed journals. The following research databases were identified by both EBSCOhost library interface websites for the review: Academic Search Complete, OmniFile Full Texas Mega (H.W. Wilson), CINAHL Ultimate, Complementary Index, and MEDLINE Complete.

### 2.2. Inclusion Criteria

Initially, the review team worked at the individual level, and then as a group, to query Google and other public search engines to identify potential search terms and/or phrases to include in the review process. Medical Subject Headings (MeSH), the controlled vocabulary thesaurus utilized for indexing articles for PubMed was also reviewed for applicable terminology, yet none identified with this review. As a result, the research team utilized Google search queries to begin establishing search terminology and related Boolean operators which yielded the highest applicable publication results around the topic. The final string identified by the research team was:

[(inflation reduction act) AND (drug*)]

The research team truncated ‘drug’ in the search string to instruct the database to search for all forms of that word (ex., drug vs. drugs), therefore yielding higher search results on both EBSCOhost platforms. This approach also worked to identify articles referring simply to ‘drugs’, as well as those referencing ‘prescription drugs’.

All articles meeting this inclusion criteria were exported from their respective research databases into a single MS Excel spreadsheet, keeping their original institution’s EBSCOhost origin database coded (Texas State University or Concordia University–Texas) for review process transparency. The spreadsheet was then sorted to identify duplicate articles identified across both searches and a single list of initial included manuscripts was recognized. This initial search string collectively yielded a combined total of 1831 results. As the Inflation Reduction Act was passed by the 117th United States Congress and signed into law by President Biden on 16 August 2022, the research team identified many IRA-related articles published prior to this date, and therefore chose to filter search results to only articles published after 1 January 2022, through 1 March 2023. On the other hand, due to prior U.S. legislation utilizing the terms ‘inflation’ and also ‘reduction’ in their policy names (to include prior Inflation Reduction Acts specifically), the research team identified other articles surrounding prior IRA-related policies prior to 1 January 2022, therefore identifying the 1 January 2022 to-date (1 March 2023) search criteria to best identify articles for this review.

### 2.3. Exclusion Criteria

Articles were included in the review if they specifically addressed the Inflation Reduction Act of 2022 and specifically addressed prescription drugs as potentially underlying themes within any identified article. Publications included in the review analysis had to initially be classified as peer-reviewed journals in the EBSCOhost search engines and meet the publication date range. Our team’s rapid review process objective was to identify any/all initial perceptions by various identified prescription drug stakeholders affected by this recent legislation, such as researchers, developers, manufacturers, insurers, dispensers, and patients. These stakeholders have immediate understanding of the pharmaceutical industry and provide assumptions and related observations from their professional position within the healthcare industry of potential outcomes which have not yet been fully identified or experienced.

**Figure 1:**
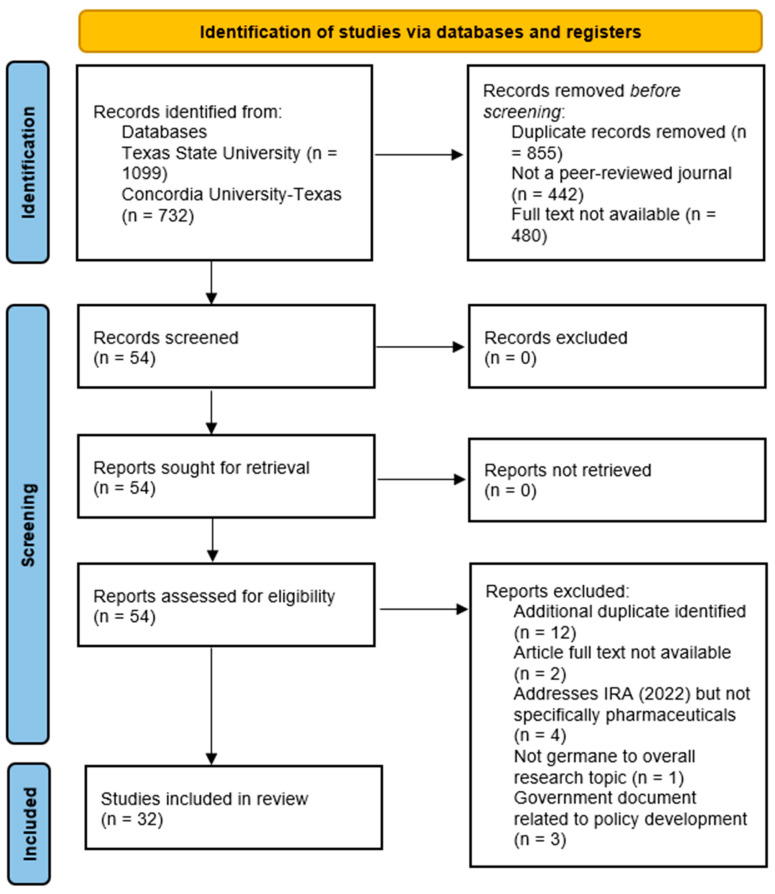
Preferred reporting items for systematic reviews and meta-analysis (PRISMA) figure that demonstrates the study selection process.

Additional search parameters were applied to produce focused, applicable results that met the team’s research objective. The EBSCOhost database platform automatically removed 855 duplicate articles. In addition to filtering for the publication date, the research team excluded any articles that were not available in full-text format (−480 articles) and were not published in peer-reviewed journals (−442 articles). All identified articles in the search were available in English, so no exclusions occurred for language filtering. A final exclusion of geography (United States only) was applied to the review findings, yet this yielded no additional articles to remove from the search. A total of 54 remaining articles were identified for this narrative review. Figure 1 illustrates the research team’s process and applied search criteria, narrowing the final number of manuscripts included in the review process to 32.

A full-text review of the remaining 32 articles was conducted by the review team, with each article being reviewed by two or more members of the research team. Table 1 shows the review assignments for the articles identified. Each article was reviewed (full-text review) by three of the research team members across all article reading assignments.

## 3. Results

The identified studies’ quality, as assessed through the use of the JHNEBP study design coding methodology, demonstrated that the majority of the literature (16 articles, 50% of the sample) falls within the level 5 category (opinions of industry experts not based on research evidence). A combined 14 (43%) articles represented JHNEBP levels 3 and 4. The remaining literature in the sample demonstrated a study design interpreted as level 2, a quasiexperimental study, or level 3, a nonexperimental, qualitative, or metasynthesis study. While the strength of evidence regarding this review’s literature sample (primarily consisting of level 4 and 5 study designs) is important to note, the researchers came to the conclusion that this observation was possibly due to the nature and timeliness of this review and ongoing publications surrounding the recent Inflation Reduction Act’s influence upon prescription drugs in the United States.

There may be limitations in carrying out true experimental studies, such as randomized controlled trials (RCTs), to evaluate the implementation of the Inflation Reduction Act (IRA) of 2022 thus far. Consequently, the research team opted for a narrative review approach, focusing on articles that were classified as level 4 and 5 evidence. This was done in an effort to gauge current opinions of various stakeholders on the subject, drawing from the available peer-reviewed literature up to this point (Table 2).

Table 2 also addresses article publication information (author(s) and article title). The table provides summaries of the stakeholder’s themselves, such as job title, place of work, etc., to assist the reader in understanding where their perspective on the IRA’s (2022) influence on prescription drugs is originating. The research team synthesized the material to provide a comprehensive summary of various stakeholder perspectives. Providing the origin of the source material, specifically identifying the stakeholders in Table 2, presents the breadth of potential bias the research team reviewed and distilled. The team identified a consistent bias from the individual groups: manufacturers were concerned with the potential impact on research and development, driven from lower revenue, insurers considered the impact on Medicare Advantage plans and cost apportionment, while dispenser and patients focused on the financial benefits and potential increased adherence to prescribed regimens.

**Table 2:** Summary of findings (n = 32).

Article Number and EBSCOhost Library Indicator († ^)	Article Title and Author Name(s)	Healthcare Stakeholder	Pricing Issue OR Dictating Pricing	Patent Law with Regard to the IRA	Process for Rules and Regulations	Threats to Pharmaceutical R&D	JHNEBP Study Design *
[1] | †	Inflation Reduction Act and US drug pricing Sarpatwari, A. (2022)	Academic (Assistant Professor)	Describes cost-saving measures at individual and federal levels involving manufacturers and insurers. The federal government must negotiate with drug manufacturers for a responsible price for brand-name drugs (initially, 10% of the highest-spend prescription drugs dispensed by pharmacies). Manufacturers will be required to issue rebates to the federal government if drug prices rise higher than the current rate of inflation. Changes to the benefit for pharmacy-dispensed drugs will occur. Insurance premiums will be limited by the Act and eliminate all out-of-pocket costs for vaccines, place a cap on out-of-pocket expenses for insulin, and expand low-income subsidies for low-income families with drug costs.		The question remains if the Medicare Advantage offering to consumer insurers recover the losses incurred from IRA on other business segments (non-Medicare).	The author states the Act is a “stinging loss for the pharmaceutical industry,” with suggestions that future legal action will stall implementation efforts.	4
[2] | †	2022 Inflation Reduction Act: Climate Investments Are Public Health Investments Levy, J. (2022)	Journal Associate Editor	The article briefly mentions the IRA’s component about the ability to negotiate drug prices within Medicare (lines 22–23). However, there is no detailed discussion or analysis of pricing issues or how pricing is dictated, particularly in relation to pharmaceuticals.	n/a	n/a	n/a	4
[3] | †	Inflation Reduction Act and the Impact on Pharmaceutical Pricing and Investment Decisions Creighton, D. (2022)	Life Sciences Consulting Firm Partner	The article discusses the IRA’s introduction of a “maximum fair price” for drugs, which could be seen as dictating pricing. This approach is described as a “take-it-or-leave-it situation” rather than a negotiation based on clinical evidence.	Addresses the implications of the Inflation Reduction Act for pharmaceutical manufacturers. It particularly highlights the impact of the Act’s reference to a “maximum fair price,” suggesting a non-negotiable situation for pricing rather than one where clinical evidence would be a key factor in determining the price.	Succinct and forward- thinking management of drug pricing will be necessary to anticipate future returns. Drug cost under the IRA reduces revenue for manufacturers.	Explores the implications of the Inflation Reduction Act for pharmaceutical manufacturers, particularly concerning the “maximum fair price” provision, and its potential impact on pricing strategies, revenue optimization, and evidence-development planning in the pharmaceutical industry. Evidence-development planning: the opportunity costs related to the return on investment for pharmaceuticals versus other commercial priorities for drug manufacturers need to be assessed with real-world data surrounding a drug’s true effectiveness. Suggests methods for drug development requires further scrutiny (e.g., new novel development or modified adjustments to existing medications).	5
[4] | †	Study: Heart Failure Patients Could Save with Inflation Reduction Act Myshko, D, (2023)	Medical Practitioner	The IRA introduces a cap on annual out-of-pocket spending for Medicare patients, which could lead to significant savings on heart failure medications. This cap effectively addresses the issue of high medication costs for patients. The author concludes by mentioning that, while often a comorbidity, heart failure patients could gain better access to these medications and possibly help address other broader medical problems, such as diabetes or cancer.				5
[5] | †	Inflation Reduction Act Could Have Ripple Effects in Medicare Part D Woody & Lazarou (2023)	Multiple Healthcare Positions, including Researcher and Healthcare Strategist	The IRA will lead to Medicare Part D plans covering costs in the catastrophic phase, but these plans are limited in how they can offset these costs due to the IRA limiting annual premium increases. This indicates a focus on controlling and potentially dictating pricing within the scope of Medicare Part D. Discusses benefits of the IRA for reduced patient payment, but queries how the funding will be generated specific to premium-increase restrictors.	Discusses the Inflation Reduction Act’s effects on Medicare Part D, particularly focusing on how the act influences Medicare patients’ out-of-pocket costs and limits annual premium increases. The article raises questions about the adequacy of new cost-sharing protections and the financial implications of these changes.		A ‘ripple effect’ identified by the author is how Medicare Part D plans are unable to increase premiums for these higher costs they will experience. This financial loss will have to be made-up by additional CMS reimbursements for sponsors to provide coverage, which will require funding for this amount for the CMS—which may be achieved by raising taxes.	5
[6] | †	Webinar: Inflation Reduction Act Will Be Positive for Medicare Myshko, D. (2022)	Not Provided	The IRA allows Medicare to negotiate prices on some drugs, limit out-of-pocket costs for Medicare beneficiaries, and cap insulin prices at USD 35 a month. This indicates a direct influence on drug pricing. Negotiated drugs are expected to save Medicare approximately USD 102 billion over six years, signifying a significant impact on pricing within the Medicare system. Changes to the drug launch prices strategy is discussed as a potential option to assist pharmaceutical companies in responding. Drug price growth beyond inflation rates is cited as still possible; however, the proposed Act will allow a much slower rate.				5
[7] | †	The Potential Impact of the IRA: Interpreting what the Inflation Reduction Act could mean for biopharma Henderson, L. (2023)	Not Provided	The IRA introduces a Drug Price Negotiation Program, allowing negotiation of a “maximum fair price” (MFP) for certain brand-name drugs without generic competitors, which affects pricing in Medicare Parts D and B. There is a suggestion that the level of penalties for not complying with the CMS rules indicates a form of direct price control, rather than negotiations.	The potential impact of the Inflation Reduction Act (IRA) on the biopharma industry is addressed. The IRA contains various parts, including drug-price negotiation, prescription drug inflation rebates, improvements in Medicare Part D, and maximum out-of-pocket caps for Medicare beneficiaries. It also mentions the possibility of the IRA being legally challenged due to its perceived nature of direct price control rather than negotiations.		The article hints at potential negative impacts on innovation, with concerns about the IRA’s effects on pharmaceutical manufacturers’ research programs and follow-on research. Challenges in selecting first drugs, price selection, opportunities for stakeholder involvement are listed in collaboration with the Pharmaceutical Research and Manufacturers of America (PhRMA). The PhRMA acknowledges the IRA's passage, but voices concerns over future design, continuing research, and specialty designation (“orphan indications”) for the development of rare conditions with decreased revenue.	5
[8] | † ^	Inflation Reduction Act Celebration Caldwell, S. (2022)	School Nurse	The IRA aims to lower prescription drug prices for Medicare, with an annual cap of USD 2000, indicating a direct influence on drug pricing for Medicare beneficiaries.		Encourages nurses to become knowledgeable regarding new policies within the IRA (2022). States that the promotion of health equity is supported by understanding and sharing health policies with patients. Important for families to be informed to be at the best advantage for health, financial, and environmental provisions (e.g., drug caps, clean tax credits). Further discussion as to how these provisions will help to address health equity, environmental justice, wealth justice, as well as other social determinants of health and social justice.		5
[9] | †	New Legislation Overhauls Medicare Drug Pricing and Benefits: Pharma loses battle to block pricing negotiations, but implementation faces many challenges Wechsler, J. (2022)	Journal Editor	The IRA will lead to significant changes in the design of drug benefits for Medicare, including capping out-of-pocket drug costs for Medicare beneficiaries at USD 2000 a year beginning in 2025. It introduces provisions for Medicare to negotiate prices on certain costly medicines, aiming to establish a maximum fair price for certain single-source drugs and biologics.	Addresses the new legislation overhauling Medicare drug pricing and benefits. It highlights the significant changes in Medicare drug benefits designed to save the government USD 100 billion over 10 years. These changes include capping out-of-pocket costs for Medicare beneficiaries, altering the Medicare Part D program, and imposing rebates or fees on pharma companies if drug prices increase beyond inflation. This article also discusses the impact of Medicare’s authorization to negotiate drug prices.	Acknowledgement of the limited understanding of the Act from pharmaceutical industry also provided.	Raises concerns about its impact on the pharmaceutical industry, particularly regarding research and development incentives, and the potential effects on generic drug makers and biosimilar manufacturers. Pharma forewarns that revenue restriction will reduce exploration and experimentation in novel drug development. Cites that some health analysts believe innovation and development will still continue, even after full implementation, due to competition and an ability to benefit Americans more broadly.	5
[10] | †	Inflation Reduction Act Contains Important Cost-Saving Changes for Many Patient—Maybe for You McAuliff, M. (2022)	Independent Reporter	The IRA allows Medicare to negotiate prices for some of the most expensive drugs, cap out-of-pocket payments for drugs, limit insulin cost-sharing to USD 35 a month, and bar drug companies from raising prices faster than inflation. The Department of Health and Human Services is tasked with identifying Medicare’s 100 most expensive drugs and then selecting 10 for price negotiations starting in 2023, with these prices taking effect in 2026.		Policy watchers are hopeful that additional programs will be influenced by the deflation of drug pricing, therefore extending to more of the population.		5
[11] | †	Pharma versus pricing, again Iskowitz, M. (2023)	Not Provided	The IRA introduces differing timelines for price negotiations for large and small therapeutics. For small-molecule drugs, prices will be negotiated nine years postapproval, while for biologics, it will be 13 years postapproval. Many in the pharmaceutical industry view these timelines as too brief to expect a reasonable return on investment, suggesting concerns about the IRA dictating pricing. States that the pharmaceutical industry is no longer fighting the existential threat to discretionary pricing power. Instead, the author states that efforts are now directed to prevent stifling of innovation in the industry. It is suggested that price negotiations will lead to a disproportionate emphasis on biologics development as a result of the shorter nine-year timeline for small-molecule treatments.	Focuses on the challenges facing the pharmaceutical industry in the wake of the Inflation Reduction Act’s drug-price negotiation provisions. It discusses how this act differentiates between the timelines for price negotiation for biologics and small-molecule drugs, the industry’s efforts to shape the new law in a way that does not hinder innovation, and potential legislative actions required to address the perceived disparities in treatment between different types of drugs.	Pharmaceutical Research and Manufacturers of America (PhRMA)’s largest concern is timeline negotiation for the small-molecule drugs, which make up 90% of the market. Compares the timeline for small-molecule and biologic drugs under scrutiny for negotiation. Proposes a longer timeline for small-molecule drugs to reassure investing stakeholders and continue unimpeded resources for continuing R&D: nine years, versus other biologics, which will be negotiated thirteen years postapproval.	There are concerns that the IRA’s price negotiation terms for small-molecule drugs, which begin much sooner, could lead to reduced investment in the development of these drugs. This might result in a disproportionate emphasis on biologics development. The industry fears that the IRA may create a long-term innovation distortion, disadvantaging small-molecule drugs and related medicines, and impacting the variety of treatments available.	5
[12] | † ^	Impending Relief for Medicare Beneficiaries—The Inflation Reduction Act Dusetzina & Haiden (2022)	Academics from the Vanderbilt University School of Medicine (S.D.) and Harvard Medical School (H.H.)	The IRA introduces limitations on increases in drug list prices to the rate of inflation and requires price negotiation for some older medications without generic or biosimilar competition. An annual out-of-pocket spending cap of USD 2000 will be added in 2025 as part of the Medicare Part D benefit redesign, which will increase each year based on the average per capita Part D spending.		Beneficiaries may be more financially inclined to begin treatment, adhere to treatment, or use higher-priced drugs rather than lower-priced options after they reach their out-of-pocket maximum. Variables surrounding how plans implement changes related to cost smoothing during early stages, as well as administrative burdens are cited as important to pharmacies. Also, the rate that the beneficiaries opt into the plan is also important. Further insight and transparency as to how plans conduct patient steerage using drug copayments is recommended.		4
[13] | † ^	Simulated Medicare Drug Price Negotiation Under the Inflation Reduction Act of 2022 Rome, et al. (2023)	Multiple Medical Providers (Physicians) and other Healthcare Professionals	The IRA includes major reforms aimed at lowering prescription drug costs: a redesign of the Medicare Part D benefit, including a USD 2000 annual limit on out-of-pocket costs, mandatory rebates for price increases exceeding inflation, and price negotiation for high-cost drugs. The CMS will select eligible drugs annually for price negotiation, with negotiated prices taking effect two years after selection. The criteria for drug selection include high Medicare spending, FDA approval duration, and the absence of generic or biosimilar competitors. The IRA instructs the CMS to negotiate a maximum fair price for selected drugs, which must fall below a ceiling price determined by either the average net price after existing rebates and discounts or a percentage of the nonfederal average manufacturer price.		Drug selection is vital, and drugs can become ineligible because generics or biosimilars might have become available.	The article suggests that the limitation of price negotiation to drugs that have been available for a certain number of years (9 years for most drugs, 13 years for biologics) might lead to delayed negotiation and could potentially affect the pharmaceutical industry’s approach to pricing and investment in new drugs.	3
[14] | † ^	What U.S. hospitals and health systems can expect from the 2022 IRA Perez, K. (2022)	VP of Healthcare Policy and Government Affairs at a Private Healthcare Firm	Medicare negotiation is a key part of the IRA’s drug pricing reforms, allowing Medicare to set the maximum fair price (MFP) for many single-source branded drugs, with the prices starting to apply in 2026 for 10 drugs from Part D. This number will increase each year, reaching a total of 100 drugs by 2031, effectively giving Medicare the power to dictate prices for these drugs. The minimum discounts that Medicare could demand for these drugs vary based on the age of the drug, with no set price floor, indicating significant control over pricing.		States that serious unresolved issues for hospitals and unintended consequences of the IRA still exist. The article concludes with a mention of no price floor to the level of discounts Medicare is able to negotiate due to the Act. Steep discounts are easily projected.		5
[15] | † ^	Bringing Transparency and Rigor to Medicare Drug Pricing Gottlieb, S. (2023)	Physician	The IRA includes provisions to lower prescription drug prices for Medicare beneficiaries, reduce out-of-pocket costs, and cut the federal government’s overall drug spending. It grants the CMS new authority to negotiate prices for small-molecule drugs that have been on the market for at least 9 years and biologics for 11 years, even if these drugs still have patent exclusivity. The law mandates that the CMS use a consistent methodology and process for establishing a price for drugs, aiming to achieve the lowest maximum fair price for each selected drug. It establishes an upper limit for the price Medicare will pay based on a percentage of a drug’s nonfederal average manufacturer price.	Focuses on the Inflation Reduction Act of 2022, detailing provisions aimed at lowering prescription drug prices for Medicare beneficiaries and reducing overall government drug spending. It grants the Centers for Medicare and Medicaid Services (CMS) authority to negotiate drug prices for certain small-molecule drugs and biologics, even if they still have patent exclusivity. The article notes that the law does not provide explicit guidelines on establishing proposed prices and exempts the program’s implementation from the formal rulemaking process, instead allowing the CMS to issue nonbinding guidance documents.	The CMS will issue nonbinding guidance documents to implement the new pricing framework, which does not require the same level of public engagement as formal rulemaking. This approach allows for more flexibility but lacks the specificity of a formal regulatory process.	The article raises concerns about the lack of a clear and consistent method for pricing discussions under the IRA, which could impact drugmakers’ decisions on allocating capital for future research and development. It suggests that understanding how federal officials price products can influence investment decisions in R&D that align with public health objectives. It highlights the new authority given to the CMS to negotiate drug prices, particularly for small-molecule drugs and biologics that have been on the market for a specified number of years, even if they still have patent exclusivity. Discusses external agencies as positive forces that take into consideration prior R&D, benefits relative to similar therapies, and the view toward best outcomes. Supports transparent and comprehensive action plan where all stakeholders can plan and anticipate for the future (e.g., pharmaceuticals and investors for R&D). Further states that, without a clear and consistent method for grounding these pricing discussions, drug companies will be challenged to make decisions about where to allocate future capital based on how public health authorities assess benefit and value.	4
[16] | ^	Out-of-Pocket Drug Costs for Medicare Beneficiaries with Cardiovascular Risk Factors Under the Inflation Reduction Act Narasimmaraj et al. (2023)	Physicians and other Healthcare Professionals	The IRA will cap Medicare out-of-pocket drug costs at USD 2,000 per year and expand full low-income subsidies (LIS). This measure is expected to alleviate financial toxicity and medication nonadherence due to high out-of-pocket prescription drug costs.				3
[17] | ^	Pharmaceutical Spending in Fee-for-Service Medicare: Looking Ahead to the Inflation Reduction Act Gellad & Hernandez (2022)	Physician; Pharmacist	The IRA represents the most transformative reform to the Medicare prescription drug benefit since its inception, introducing measures such as manufacturer rebates for price increases above inflation, negotiation of prices for high-spending drugs, and a cap on Part D out-of-pocket expenses.				4
[18] | ^	The Inflation Reduction Act: A boon for the generic and biosimilar industry Niazi, S. (2022)	Academic (Pharmacy School)	The IRA aims to reduce the burden of Medicare by over USD 100 billion per year, focusing on a fixed number of top expenditure drugs that have remained as single-source chemical products for 8 years and biologics for 12 years. The number of products negotiated for price reduction will increase from 10 to 20 over the years. The IRA includes provisions for Medicare to directly negotiate prices with pharmaceutical manufacturers for certain high-spend Medicare drugs, with penalties for companies that refuse. It also establishes an annual out-of-pocket cap for beneficiary cost-sharing on prescription drugs.	The article discusses the IRA’s legislative framework and the analysis of related statutes in consultation with legal teams. However, it does not provide detailed information on the specific processes for establishing rules and regulations under the IRA. Discusses the impacts of the Inflation Reduction Act on biosimilar manufacturers and the pharmaceutical market. It addresses concerns about the reduction in prices for biological drugs after their exclusivity periods and the viability of biosimilar products in the market. The article also discusses the misconceptions related to the negotiation process, monopoly dynamics, and the impact of the IRA on the private market. Moreover, it touches on issues related to biologic brands and patenting practices, as well as the overall effectiveness of the Inflation Reduction Act in fostering competition and sustaining lower prices.	Reaching any significant number out of the 14,000 reimbursed drugs will take forever if biosimilars and generics continue to be entered the approved reimbursement formulary. The IRA includes restrictions to prevent the brand-name companies from exploiting the entry of generics and biosimilars to assure their independence.		3
[19] | ^	Assessing US Pharmaceutical Policy and Pricing Reform Legislation in Light of European Price and Cost Control Strategies Rodwin, M. (2022)	Academic	The United States pays more for identical branded drugs than European nations, with per capita spending on prescription drugs more than double that of the UK, France, and Germany. This price difference has increased over time. The article attributes this to U.S. policies and those proposed in recent bills, including the IRA, and compares them with European cost-control strategies.	One of the main reasons for high prescription drug prices in the United States is patents and FDA grants of market exclusivity, which create an effective monopoly, allowing manufacturers to set prices. The article discusses this issue in the context of U.S. policies and recent legislation, including the IRA. Further reviews the Inflation Reduction Act (IRA) and the Build Back Better Act (BBBA), focusing on their approach to drug pricing negotiations. It compares the timing of these negotiations with practices in European countries, highlighting differences in capping prices and addressing price increases. The article also discusses the absence of legislation in the U.S. that prohibits charging uninsured individuals’ higher prices than the insured, and the complexities involved in adopting European-style drug-pricing controls in the U.S. due to various political and institutional barriers.	Authors call for price negotiations to be transparent and rigorous.		4
[20] | ^	Federal Officials Issue Initial Guidance to Rein in Drug Spending Harris, E. (2023)	Not Provided	The plan aims to decrease drug prices under the IRA. It includes the Medicare Prescription Drug Inflation Rebate Program, which requires companies to pay rebates to Medicare if they increase their prices for both Part B and Part D drugs faster than the rate of inflation.		Brief mention of the CMS plans to enforce the IRA rebate provision.		5
[21] | ^	The Inflation Reduction Act: Recasting the Medicare Prescription Drug Plans Adashi, et al. (2023)	Not Provided	The IRA amends the noninterference clause, allowing the Secretary of Health and Human Services (HHS) to negotiate drug prices for Medicare Part B and Part D. Negotiations over the price of the 10 most costly Part D prescription drugs lacking generic or biosimilar competitors will begin in 2026, increasing to 15 drugs by 2027 and 2028, and 20 drugs annually thereafter. The IRA caps the out-of-pocket pharmacy expenditures of Medicare Part D enrollees at USD 2000 per year starting in 2025 and caps the out-of-pocket copayments for insulin by Medicare beneficiaries at USD 35 per month starting in 2023.	Examines the Medicare Prescription Drug Improvement and Modernization Act of 2003 (MMA) and the Inflation Reduction Act’s impact on rescinding the noninterference clause in the MMA. It discusses President Biden’s support for legislative reforms to lower prescription drug prices, estimates from the Congressional Budget Office on the fiscal impact of these reforms, and various aspects of the IRA, including caps on out-of-pocket pharmacy expenditures and impacts on Medicare spending on prescription drugs. The article also considers the potential challenges in implementing these policy changes, including legal challenges and the influence on future Congressional interest in drug pricing reforms.	Article reviews the projected attenuation of the national prescription drug bill and assumes that the maximum fair price arrived at by the negotiating parties will be applied to Medicare Part B and D plans and enrollees. Authors call for price negotiations to be transparent and rigorous.	The IRA could be subject to legal challenges on the part of the pharmaceutical industry as well as to legislative repeal effort.	5
[22] | ^	Estimated Medicare Part B Savings from Inflationary Rebates Egilman & Kesselheim (2023)	Academics (Program on Regulation, Therapeutics, and Law)	The IRA includes a provision requiring prescription drug manufacturers to pay rebates to Medicare if they raise prices faster than inflation. This policy aims to protect Medicare from excessive price increases by manufacturers, as from 2007 to 2018, net drug prices had outpaced inflation by an average of 4.5% per year. The article includes a simulation of this policy’s application to Medicare Part B if the rebates had been in effect from 2018 to 2020.	Addresses the current patent and regulatory system’s impact on pharmaceutical innovation. It highlights how minor changes to the delivery systems of existing molecules are rewarded, potentially diverting incentives away from new therapeutic breakthroughs. The article notes that regulators and lawmakers have begun scrutinizing patenting practices related to drug–device combinations, suggesting a need for substantial reform to prevent continued high spending on products with long-established active ingredients.			3
[23] | ^	The Inflation Reduction Act Will Change Who Pays for Cardiovascular Drugs under Medicare Part D Kazi et al., 2023	Medical Providers and Medical School Academics	The IRA will cap out-of-pocket drug costs at USD 2000 for 48 million Medicare Part D enrollees starting in 2025. This change is expected to significantly alter the distribution of drug costs, with patients and Medicare bearing a lower share, and plans paying a larger share. Under the IRA, patients will pay ≤25% of the total drug costs and Medicare’s share of drug costs will be capped at 20%, but plans’ share of drug costs would rise substantially to ≥60% for the 4 CV conditions examined.		Researchers examined how payers’ share of drug costs will change with the IRA for 4 cardiovascular (CV) regimens.	Suggests that efforts to actively manage prices will stifle innovation.	3
[24] | ^	Projected Impact of the Inflation Reduction Act on Out-Of-Pocket Drug Costs for Medicare Part D Beneficiaries with Cardiovascular Disease Kazi, et al., 2023	Medical Providers and Medical School Academics	The IRA redesigns the Medicare Part D drug benefit for its 48 million beneficiaries by eliminating catastrophic coinsurance and capping cost-sharing. This is projected to significantly reduce OOP costs for key CV drugs. The IRA will lower OOP costs for CV conditions. Patients with the costliest regimens (e.g., for amyloidosis) will see the largest savings starting in 2024. Patients with all 4 conditions examined will have lower OOP costs in 2025, which may alleviate cost-related nonadherence.			The IRA will lower OOP costs for CV conditions. Patients with the costliest regimens (e.g., for amyloidosis) will see the largest savings starting in 2024. Patients with all 4 conditions examined will have lower OOP costs in 2025, which may alleviate cost-related nonadherence.	3
[25] | ^	Medicare Drug Price Negotiation in the United States: Implications and Unanswered Questions Sullivan, S. (2023)	Academic (School of Pharmacy)	The United States has historically been a free-pricing market for pharmaceutical manufacturers to set list prices at product launch. The IRA introduces direct price negotiation between Medicare and manufacturers, representing a significant change in the U.S. market. This development is seen as a watershed moment for addressing high drug prices and their impact on Medicare budgets and patient out-of-pocket costs.			A key concern regarding the IRA’s impact on drug pricing is the potential to stifle innovation. Critics argue that actively managing prices could reduce revenue for the pharmaceutical industry, which in turn might lead to a reduction in R&D activity and innovation. Estimates vary on the potential reduction in the number of new drugs developed as a result of the IRA’s pricing negotiations. Returns on substantial R&D costs will be affected, as will future investments, but how companies choose to manage and redirect fewer resources remains a question. Price negotiations on selective drugs only reduce the spending by about 5.4 percent and may not affect the pharmaceutical industry as much as thought. This would change if more drugs were added to the negotiation list as pharma fears. There is a little doubt that lower revenue as a result of price negotiations will affect research and development.	3
[26] | ^	Medicare Negotiation of Prescription Drug Prices Ubl, S. (2022)	CEO of a Pharmaceutical Trade Group	Since there is no price floor in the IRA, Medicare could impose discounts. As for 340B, launch prices could well go up, and the 340B price negotiations, which are not controlled by the IRA, could inhibit the lowering of prices under 340B not controlled by the IRA.	To recoup lost revenues caused by the caps and, eventually, the Medicare-imposed discounts on the most successful drugs, drugmakers would likely increase drug launch prices.			5
[27] | ^	New Reforms to Prescription Drug Pricing in the US: Opportunities and Challenges Hwang, et al. (2022)	Academics (Pharmacy School)	The IRA allows Medicare to directly negotiate prices for a limited number of brand-name drugs with the greatest spending under Part B (clinician-administered drugs) and Part D (retail drugs). This negotiation starts with 10 drugs annually in 2026, increasing to 20 drugs annually by 2029. The legislation also imposes a penalty on manufacturers that increase drug prices faster than inflation, starting in October 2022. This expansion includes rebates to Medicare Part B and Part D based on Medicare sales and price increases exceeding inflation for new and existing drugs since January 2021.	Authors state that it is highly likely that many of the top-selling drugs in the Medicare program will qualify for negotiation under the Inflation Reduction Act, because the average length of time before entry of generic or biosimilar competition has historically exceeded 14 years.	Implementation of these drug-pricing reforms will be important.		4
[28] | ^	Landmark law to curb Medicare drug prices will start to make an impact in three years Hut, N. (2022)	Journal Editor	The Inflation Reduction Act mandates that Medicare negotiate the prices of certain drugs, aiming to reduce both out-of-pocket costs for beneficiaries and federal healthcare spending. This landmark legislation is expected to significantly impact Medicare drug spending.	The negotiated prices under the Act will start in 2026 for 10 Medicare Part D drugs, as selected by the U.S. Department of Health and Human Services (HHS). More drugs will be added in subsequent years, with Part B drug prices becoming eligible for negotiation in 2028.			5
[29] | ^	Biotech Burnout Eisberg, N. (2022)	Journal Editor	The Inflation Reduction Act, forming part of the Senate’s Prescription Drug Pricing Bill, introduces negotiations between the pharmaceutical industry and Medicare. This act includes provisions that are expected to come under intense scrutiny, particularly concerning how they will affect the pharmaceutical sector.	So-called ‘negotiations’ are really a smokescreen behind which Medicare can enforce financial punishments on companies that do not comply with its pricing proposals.	The U.S. Department of Health and Human Services is tasked with generating the necessary regulations to enact the requirements of the Act. This indicates that detailed processes for establishing rules and regulations are still in development.	Some biotech investment firms have expressed concerns that the bill could make the development of small-molecule drugs ‘uninvestable’. Additionally, the Pharmaceutical Research and Manufacturers of America (PhRMA) and individual drug companies are preparing to respond to what they perceive as enforced financial punishments for not complying with Medicare’s pricing proposals under the guise of negotiations. Numerous biotech firms are laying off employees.	5
[30] | ^	Congress Extends Enhanced ACA Subsidies Keith, K. (2022)	Academic (Health Law)	The IRA introduces historic reforms, notably including Medicare prescription drug negotiation, which is a significant shift in health policy. This reform is part of a broader package of measures in the IRA aimed at addressing various aspects of healthcare and the economy.	Covers the Inflation Reduction Act, focusing on its major health reforms, including the extension of enhanced marketplace subsidies and historic Medicare prescription drug negotiation reforms. It details the new law’s significant investments in healthcare, including caps on overall out-of-pocket drug spending for seniors, and a copay cap for insulin products. Additionally, the article discusses significant court decisions impacting health policy, such as a Texas judge’s ruling on parts of the ACA’s preventive services mandate and the revised nondiscrimination rule issued by HHS under the Biden administration, affecting health insurers and healthcare decision-making.			5
[31] | ^	Estimates of Insulin Out-of-Pocket Cap– Associated Prescription Satisfaction: Adherence, and Affordability Among Medicare Beneficiaries Li, et al. (2023)	Academics (Schools of Pharmacy)	The IRA caps insulin out-of-pocket costs at USD 35 per month, which is expected to benefit nearly half of Medicare-insured insulin users. This policy change is likely to improve prescription satisfaction, adherence, and affordability among patients.	Researchers examined the association of the insulin out-of-pocket cap with prescription satisfaction, adherence, and affordability among Medicare-insured insulin users and to identify associated disparities. Findings suggest the cap on insulin out-of-pocket costs at USD 35 per month will likely benefit half of Medicare-insured insulin users.			2
[32] | ^	The Inflation Reduction Act’s Out-Of-Pocket Prescription Drug Cost Cap Will Benefit over 1 Million Medicare Patients with Cardiovascular Risk Factors or Disease Narasimmaraj, P. et al., 2023	Cardiology Faculty Fellow; Various Healthcare Professionals and Researchers	The IRA, passed in August 2022, will limit annual OOP drug costs to USD 2000 for all Medicare Part D beneficiaries. This policy is particularly relevant for adults with CV comorbidities, who often face high financial burdens from OOP drug costs.	Findings include an estimated 39.5 million U.S. adults ≥ 65 years had Medicare Part D coverage, of whom 1.1 million (2.8%) had annual OOP prescription drug costs > USD 2000. Researchers determined the IRA′s USD 2000 annual limit on OOP prescription drug costs will benefit 1.02 million patients with CV comorbidities annually.			2

† Manuscript was identified via the Concordia University–Texas library EBSCOhost website. ^ Manuscript was identified via the Texas State University library EBSCOhost website. * Johns Hopkins Nursing Evidence-Based Practice (JHNEBP) levels of strength of evidence: Level 1, experimental study/randomized control trial (RCT); Level 2, quasiexperimental study; Level 3, nonexperimental, qualitative, or metasynthesis study; Level 4, opinion of nationally recognized experts based on research evidence/consensus panels; Level 5, opinions of industry experts not based on research evidence. Construct Code identification: A: pricing issue or dictating pricing; B: patent law with regard to the IRA; C: process for rules and regulations; D: threats to pharmaceutical R&D.

**Figure 2:**
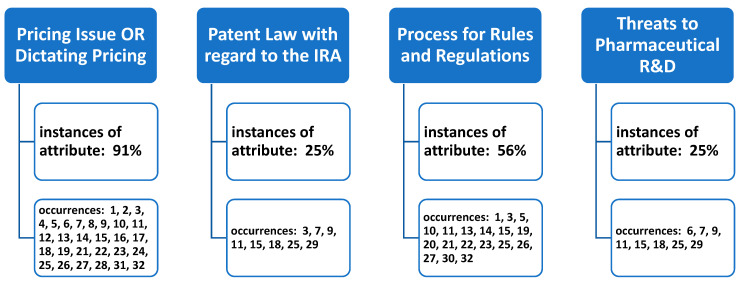
Occurrences of underlying themes (constructs) identified in the literature of stakeholder perspectives of the Inflation Reduction Act’s (2022) impact on prescription drugs.

Once the total of thirty-two articles were reviewed by the research team, four underlying constructs (themes) were identified in the review. The research team met via webinar on multiple occasions to address the underlying constructs identified in the article reviews, and agreement was reached on the themes surrounding the stakeholder perspectives on the IRA’s (2022) influence on prescription drugs in the U.S. Article inclusion into thematic categories was not mutually exclusive, with any single article in the review often meeting criteria to be coded and assigned under more than one review theme (Figure 2).

## 4. Discussion

### 4.1. Pricing

The historic IRA provides healthcare policy makers and the federal government new empowerment to deliberate the pricing of pharmaceuticals, approximating drug-cost policies that exist in European nations due to their freedom from patent protection constraints [19]. This new era of cost control allows the leveraging of lower drug costs for Americans on Medicare by complex and myriad forms.

Beginning in 2025, Medicare recipients will secure an out-of-pocket (OOP) pharmaceutical cap of USD 2000 annually, and indexed annually thereafter [33]. Drug costs above the cap will be absorbed by the insurers at 60%, and both Medicare and the manufacturers at 20%. Additional restraint on insurance premiums is also targeted to curtail costs [1,9].

The next inflationary and controversial measure is set for 2026, with the ten highest-spend pharmacy-dispensed drugs establishing a “maximum fair price” (MFP) arbitrated by Medicare and the manufacturers. The continuance of cost saving for patients will extend the MFP to twenty of the highest-spend pharmaceuticals in 2029 [1,3].

Additionally, drug prices that increase over the rate of inflation will be subject the manufacturers to furnish rebates to Medicare, further protecting older Americans from escalating medication costs. Further provisions for saving are waivers for vaccine costs, caps for insulin payments at USD 35 per month, and increasing aid for low-income individuals, thereby facilitating medication availability and compliance [1,6,10,12,16,24,31].

It is the communicated position of patient advocacy and healthcare providers, such as the Association of American Medical Colleges (AAMC), to endorse the changes the IRA will make on the financial and physical well-being of patients, whereby the ability to obtain and maintain drug coverage will be eased, saving consumers millions of dollars [6,12,14]. In a simulated study [13], Medicare benefited by saving millions of dollars with drug-cost negotiation with the manufacturers. However, Medicare will also need to source funding for the 20% of drug costs that rise above a patient’s annual cap and are dually restricted by the reduced premiums for Part D [1,5,9,12].

On the other hand, as patients and communities reap the benefits of reduced costs, pharmaceutical companies are threatened by the loss of control and revenue of selected first and subsequent drugs. The forfeited monies due to negotiation, coupled with hovering penalties [1,9], have the Pharmaceutical Research and Manufacturers of America (PhRMA) cautioning that research and development will be adversely affected [7,9,11,25]. Concentration on drug selection, “optimal launch price”, and timelines for negotiation are of foremost importance to the PhRMA [3,6,7,26], and the prospect of legal or legislative repeal or challenge to the IRA by the pharmaceutical companies cannot be wholly dismissed [1,7,21].

Policymakers hold the middle ground, theorizing that competition will advance experimentation and progress will continue in drug innovation despite reduced revenue [9]. Another hope is that, by decreasing drug prices in Medicare, Medicaid and commercial plans may eventually find reprieve from escalating drug costs [1,9,10]. The one perspective most stakeholders agree on is the challenge and importance of drug selection, and that the process be transparent and consistent [3,7,13,15,34].

### 4.2. Patent Law

While the predominance of the material reviewed focused on the effects of pricing to consumers and the reimbursement the CMS would negotiate, there were conflicting viewpoints from pharmaceutical representatives and industry analysts. Primarily, the impact of the IRA on pharmaceutical the industry will potentially lead to a change in portfolio management, specifically the mechanics of portfolio valuation and strategy regarding the return on investment (ROI), as well as the ownership or foregoing of exclusivity in favor of the early entrance of generics [3,9,18]. Further, and similar to the 1984 Hatch–Waxman Act, which resulted in the introduction of generics and overall cost reduction, the pharmaceutical industry adjusted to the new norms, as shown by 90% of all dispensed drugs being generics while only representing 15% of the reimbursement [18]. While the IRA is presented as a new approach for government-sponsored healthcare programs, in fact, the Veterans Administration has historically negotiated price and, as of 2020, paid 54% less than Medicare Part D programs [25]. The negative impact on drug patents is noted primarily in material from the industry and proxy agencies representing pharmaceuticals, describing either financial or strategic choices that would limit drug research and intellectual property [3,7,9,11,25]. Conversely, academics and practitioners argue the alternatives to limiting the effects on patents [15,18,25]. The opposition regarding the effect on patents and intellectual property strategy addresses the potential financial impact of the IRA.

The Inflation Reduction Act’s impact on the future earnings of the pharmaceutical industry concerns Stephen Ubl, President of the Pharmaceutical Research and Manufacturers of America (PhRMA), and Ed Schooveld, managing partner of ZS, a value and access practice [7]. What is implied, and not discussed, is the potential financial impact immediately upon the selection and subsequent negotiation under the Act. While patent strategies are discussed, identifying potential drug therapies that may be abandoned midstream or not presented for development, the selection by the CMS does not include future nonmonetized therapies. The reduction in future revenues would trigger an accounting valuation change on the patent value. The potential intangible asset value could result in a reduction or the impairment of the patents, in the case relating to existing portfolios, and potential immediate financial losses, as per the Financial Accounting Standards Board (FASB). FASB rule 350-30-35—14 [1] defines the treatment of both the value of, and the impairment of, intangibles, which includes patents. Through the negotiation of the “maximum fair price”, the future revenue streams connected to the asset potentially could lower the fair value below that of the balance sheet, triggering an impairment and immediate expense [34]. With a 12% success rate of products under development entering the market beyond clinical trials, and the USD 1–2 billion cost to develop a new drug, the additional impact of the impairment on existing and profitable products could have a depressing effect on the return on investment that shareholders are seeking [11].

The Act does provide avenues from which the pharmaceutical companies can avoid the impact of the impairment. The patent exclusivity timeframe is impacted by the passing of the IRA for drugs selected for negotiation, but the foregoing of patent exclusivity in favor of the stimulation of generic products could present an alternative. Under the IRA, drugs which are expected to have a generic entrant to the market within two years are excluded from its scope [18].

### 4.3. Process for Rules and Regulations

The identified literature in the search clearly demonstrated the IRA (2022) was enacted in response to rising pharmaceutical costs that have burdened American citizens for years. The legislation aimed at making prescription drugs more affordable and accessible, while also ensuring that the quality and innovation in the industry remain uncompromised. A primary theme deduced from the research team’s manuscript review entailed overall stakeholder discussion surrounding the Act’s specific process and rules for regulations and related implementation initiatives. The IRA’s influence on prescription drugs remains widespread, to include the following main process changes to Medicare Part D [3,5,19]:

- Canceling the 5% coinsurance for catastrophic coverage;
- Expanding eligibility for financial support/assistance;
- Limiting premium increases from 2024 to 2029;
- Allowing Medicare to negotiate drug prices on behalf of consumers with pharmaceutical companies.

The review team focused on those identified subconstructs related to this theme as identified only in the review literature (exclusive only to this review’s findings).

#### 4.3.1. Increased Drug Price Transparency

Central to the Act’s changes was a mandate for increased transparency in drug pricing for those medications selected for price negotiation [23]. Pharmaceutical companies are now required to disclose the breakdown of research, development, production, and marketing costs for each drug, allowing consumers and regulators a much clearer view into the factors driving drug prices. Further, and relevant to current/ongoing economic inflation challenges in the United States and beyond, a new provision prevents drug manufacturers from raising prices beyond a specified inflation rate without a justified reason, and establishes even more transparency in drug pricing [12,18]. This move was taken to prevent arbitrary price hikes, which have previously left patients unable to afford their necessary medications.

#### 4.3.2. Drug Patent System Changes

Additional market forces and the CMS review may impact the value and monetization of patents moving forward [19]. This was completed to promote competition and reduce monopolistic practices; the new regulations restrict the duration and extent of exclusivity for certain drug patents, especially for medications that are crucial for public health [11,19,21]. While the IRA does not directly affect drug patents, it may impact the future revenue from new therapies by either reducing or limiting the length of time manufacturers can realize exclusive earnings. These barriers would further ensure that generic versions of essential drugs come into the market faster, providing more affordable options for consumers [19,21].

#### 4.3.3. Pharmaceutical Negotiation Practices

The Act also establishes a body to negotiate drug prices directly with pharmaceutical companies on behalf of government healthcare programs, specifically Medicare [10,11,14,15]. This is expected to leverage the government’s buying power to drive down prices for millions of beneficiaries. The results of these negotiations would also be made public, bolstering the Act’s overarching goal of enhancing transparency and accountability in the pharmaceutical sector [14,15]. The concept of ‘launch pricing’ was also identified by the research team. The change in launch price, or market entry pricing during initial exclusive periods, may assist pharmaceutical companies in subsequent price negotiations with government and other agencies when the drug falls under the scope of price negotiation. By establishing a higher beginning drug price to begin negotiating [6], the starting negotiations benefits the pharmaceutical company, as opposed to beginning negotiations at a lower market launch price [6].

Cost-effectiveness analysis (CEA) has potential applications in setting and negotiating launch prices for medications under the Inflation Reduction Act, particularly within the context of Medicare drug-price negotiations. However, a significant challenge in using quality-adjusted life years (QALYs) in economic analyses for these purposes is legislatively restricted. The Act explicitly prohibits the use of evidence from the CEA that would treat extending the life of an elderly, disabled, or terminally ill individual as of lower value than that of someone younger, nondisabled, or not terminally ill. In negotiating the “maximum fair price” for drugs, the Centers for Medicare and Medicaid Services (CMS) must consider a variety of factors, including manufacturer-specific data, such as research and development costs, production and distribution costs, federal financial support, and market data. Additionally, the CMS evaluates information about therapeutic alternatives, including the comparative effectiveness and how the selected drugs and alternatives meet the needs of specific populations, without employing QALYs.

To establish initial offers for fair prices, the CMS identifies therapeutic alternatives and uses their pricing as a starting point, adjusting based on the clinical benefits and manufacturer-specific data. This process includes a review of a wide range of evidence on clinical benefits relative to therapeutic alternatives and patient-centered outcomes. The integration of these elements in pricing discussions reflects the complex considerations involved in price negotiations that aim to balance cost with therapeutic value.

### 4.4. Threats to R&D

The IRA has been characterized as an impediment to the development of new therapies reimbursed through Medicare Parts B and D, while the cost of development and regulatory approval remains consistent pre- and post-IRA. The selection of potential therapies will therefore be tailored to meet the new market dynamics. While the Act provides a general description of how the MFP will be conducted, there is also a requirement that a “consistent methodology and process” be utilized [15]. This process has been hard to understand and has led to some uncertainty [15]. The long-term impact in global markets [25], such as the EU seeking similar historical discount rates from the CMS, and of private payors following the CMS methodology [18,25], could lead to long-term research and development distortion [11].

The uncertainty of the negotiated MFP will impact the future decisions made by the pharmaceutical industry, which spent USD 83 billion on R&D in 2019. [25] With a high failure rate and high costs associated with bringing a drug to market [3], the decision to proceed with research will be focused on perceived longer-term profitable projects. The effect of the IRA may influence the decision at which to proceed with early-stage assets [25], leading to higher rigor in supporting research for successful advancement.

Starting in 2026, the ten drugs first selected under the IRA will be subject to “fair market prices” (FMPs). At first, the FMPs will apply to prescription drugs under Medicare Part D, but in 2029, the FMPs will also apply to hospital-administered drugs and infusion settings outside of hospitals under Medicare Part B. These selected drugs must have been approved by the Food and Drug Administration (FDA) or licensed for at least seven years (eleven years for biologicals or large molecules), not compete with a generic or cheaper version, and not qualify as an orphan drug (treating a condition that affects fewer than 200,000 people). It is widely assumed the FMPs will put downward pressure on the prices of drugs outside of the IRA coverage, with prices now largely controlled by private insurers. As the IRA 2022 impacts the revenue of pharmaceutical manufacturers by reducing the FMP to a negotiated level, they will bear the burden of the cost reduction as the passthrough to pharmacies and patients within Part D. To mitigate the reduction in revenue, the pharmaceutical manufacturers would seek to isolate their product lines under the established exclusions.

Pharmaceutical companies believe the negotiations called for in the IRA will result in dictated prices, even though they are supposed to consider a drug’s clinical benefit, the extent it fills an unmet medical need, its impact on people who depend on Medicare, and the research and development costs. Companies have filed lawsuits to make the process of setting FMPs illegal as a form of price-fixing [15]. Several Constitutional issues have been raised. Meanwhile, the process of setting FMPs has already begun, and companies will have to participate in negotiation, or their drugs can be banned from use in the Medicare program.

Although patient advocates have won price controls, their success might be short-lived. Commentators have identified several unintended consequences of the IRA:

- The FMPs might discourage generic drug makers from offering knockoffs of the branded drugs, but may also increase the early access granted by the patent holder [15].
- Companies will favor biologics (large molecules) for development over small molecules because they will have longer to earn back the development costs; they will have eleven years rather than the seven years for small molecules [35].
- Companies will have incentive to set higher launch prices to recoup as much development costs as possible before the drug becomes subject to the FMP.
- Some drugs are approved by the FDA at first for use by a small group of patients, such as drugs for rare or late-stage cancers. Approval for a more general use can be delayed for several years. The period for a company to control its own prices before the drug comes under an FMP starts on the date the drug is approved by the FDA for first use. The IRA tends to encourage companies to seek approval for wider use first to maximize return.
  ○ For example, a pharmaceutical company could postpone the launch of a drug for ovarian cancer in favor of its first approval for prostate cancer because the latter is a much larger market.
- Drug company return on investment is unpredictable, because bringing a drug to market often costs more than a billion dollars. Further billions are spent on drugs which are ultimately found ineffectual. It stands to reason that companies will reduce the overall investment out of concern for reduced income due to price controls. Already, a drug company has abandoned further development of a small-molecule cancer drug, citing the IRA as a reason [36]. The belief that drug companies make much higher profits than other companies is disputed. It depends on whether drug-development costs are treated as investments or expenses [36].

## 5. Conclusions

This narrative literature review has unveiled several prominent themes that underscore the multifaceted impact of the Inflation Reduction Act of 2022 on the landscape of prescription drugs. Analysis of recent literature has highlighted a consistent narrative surrounding the Act’s potential to mitigate rising drug costs by implementing pricing regulations and fostering competition within the pharmaceutical market. Additionally, we identified a theme emphasizing the Act’s potential in improving the affordability and accessibility of prescription drugs for patients, addressing a critical concern in contemporary healthcare. Further examination of the literature revealed nuanced discussions regarding the Act’s role in balancing the interests of various stakeholders, including pharmaceutical companies, healthcare providers, insurers, and patients, while striving for equitable and sustainable healthcare solutions. These identified themes collectively provide crucial insights for policymakers, advocating for informed decision-making and further research to maximize the positive impact of the Inflation Reduction Act and other similar policy interventions in the healthcare sector. Further topics of inquiry and of importance, while not identified in the review, surround the implications and/or challenges with pharmacy benefit managers, the potential spillover effect of negotiated pricing on private insurance plans or hospital purchasing, as well as the potential for insurance companies to exit Medicare Part D due to the limitations on insurance premiums [37]. Future empirical studies may also be analyzed in the future to potentially align identified themes with categories of healthcare stakeholders.

As with any narrative literature review, limitations do exist. As a very current, developing topic, stakeholder perceptions of the IRA’s impact on prescription drugs will continue to change. Continuous review of the literature is required, and future study is required in this regard. Another study limitation is the lack of data-driven, empirical studies included in the review findings. However, the review topic (primarily stakeholder perceptions), industry expert opinion, and healthcare leader views required inclusion of a variety of literature beyond typical research studies.

## Figures and Tables

**Table 1:** Reviewer assignment of the initial database search findings (full article review).

Article Assignment	Reviewer 1	Reviewer 2	Reviewer 3	Reviewer 4
Articles 1–10	X	X	X	
Articles 11–20	X	X	X	
Articles 21–32	X	X		X

## Data Availability

Not applicable.
